# Hemolytic, anticancer and antigiardial activity of *Palythoa caribaeorum* venom

**DOI:** 10.1186/s40409-018-0149-8

**Published:** 2018-04-17

**Authors:** Fernando Lazcano-Pérez, Ariana Zavala-Moreno, Yadira Rufino-González, Martha Ponce-Macotela, Alejandro García-Arredondo, Miguel Cuevas-Cruz, Saúl Gómez-Manzo, Jaime Marcial-Quino, Barbarín Arreguín-Lozano, Roberto Arreguín-Espinosa

**Affiliations:** 1 Departamento de Química de Biomacromoléculas, Instituto de Química, Universidad Nacional Autónoma de México, Av. Universidad 3000, Ciudad Universitaria, C.P. 04510. Apdo, Postal 70250 Mexico City, Mexico; 2 Laboratorio de Parasitología Experimental, Instituto Nacional de Pediatría, Insurgentes Sur 3700-C, 04530 Mexico City, Mexico; 3 Laboratorio de Investigación Química y Farmacológica de Productos Naturales, Facultad de Química, Universidad Autónoma de Querétaro, Centro Universitario, 76010 Querétaro, Mexico; 4 CONACYT-Instituto Nacional de Pediatría, Secretaría de Salud, 04530 Mexico City, Mexico; 5 Laboratorio de Bioquímica Genética, Instituto Nacional de Pediatría, Insurgentes Sur 3700-C, 04530 Mexico City, Mexico

**Keywords:** Cnidarian, *Palythoa caribaeorum*, Cytotoxin, Antitumoral effect, Giardiasis

## Abstract

**Background:**

Cnidarian venoms and extracts have shown a broad variety of biological activities including cytotoxic, antibacterial and antitumoral effects. Most of these studied extracts were obtained from sea anemones or jellyfish. The present study aimed to determine the toxic activity and assess the antitumor and antiparasitic potential of *Palythoa caribaeorum* venom by evaluating its in vitro toxicity on several models including human tumor cell lines and against the parasite *Giardia intestinalis*.

**Methods:**

The presence of cytolysins and vasoconstrictor activity of *P. caribaeorum* venom were determined by hemolysis, PLA_2_ and isolated rat aortic ring assays, respectively. The cytotoxic effect was tested on HCT-15 (human colorectal adenocarcinoma), MCF-7 (human mammary adenocarcinoma), K562 (human chronic myelogenous leukemia), U251 (human glyoblastoma), PC-3 (human prostatic adenocarcinoma) and SKLU-1 (human lung adenocarcinoma). An in vivo toxicity assay was performed with crickets and the antiparasitic assay was performed against *G. intestinalis* at 24 h of incubation.

**Results:**

*P. caribaeorum* venom produced hemolytic and PLA_2_ activity and showed specific cytotoxicity against U251 and SKLU-1 cell lines, with approximately 50% growing inhibition. The venom was toxic to insects and showed activity against *G. intestinalis* in a dose-dependent manner by possibly altering its membrane osmotic equilibrium.

**Conclusion:**

These results suggest that *P. caribaeorum* venom contains compounds with potential therapeutic value against microorganisms and cancer.

## Background

The phylum Cnidaria comprises approximately 11,000 species classified into seven classes (Anthozoa, Scyphozoa, Cubozoa, Staurozoa, Polypodiozoa, Myxozoa and Hydrozoa) [1]. All of them are considered to be toxic [2]. Moreover, some of them have been reported to be capable of causing severe intoxication by stinging with their specialized organelles called nematocysts [3]. Extracts of cnidarian tissues have been found to contain a complex mixture of low molecular weight compounds, peptides and proteins that together cause the paralysis and envenomation of their prey or predator [4, 5].

Venoms isolated from almost all classes of cnidarians have been found to be cytotoxic in several cellular or animal models [6]. Among the best known cytotoxic venoms are the Portuguese man-of-war hydrozoan *Physalia physalis*, the box jellyfish *Chironex fleckeri*, the jellyfish *Pelagia noctiluca*, the fire coral *Millepora complanata* and many sea anemones extracts [7–14]. Due to the wide range of biological activities of these venoms, many substances isolated from them, especially those derived from sea anemones, have served as useful molecular models and probes in biomedical research [15]. However, the antimicrobial activity of such extracts has been little explored. A few reports can be found in the literature about the antiparasitic and antibacterial properties of some cnidarians and even an antimicrobial peptide isolated from *Aurelia aurita* has been sequenced [16, 17].

Zoanthids (order Zoantharia, class Anthozoa) are organisms commonly found in shallow zones of coral reefs. This group of cnidarians has not been extensively studied as other cnidarians such as sea anemones or jellyfish. Some biochemical and toxicological research on zoanthids have proved that they possess compounds with biological activity. For instance, the existence of palytoxin, one of the most potent marine toxins known to man and first isolated on a zoanthid of the gender *Palythoa*, later discovered to be synthesized by dinoflagellates [18, 19]. Besides palytoxin, not many studies on the biological activity of zoanthid venoms or toxins have been characterized to date. An extract of their soft tissues was tested for antibacterial activity and it was found that it inhibits *Escherichia coli* and *Staphylococcus aureus* in 97.7 and 100%, respectively [20]. More recently, *P. caribaeorum* extracts were found to have antioxidant effects and cytotoxic activities [21].

According to Suput [15], an assessment of the pharmacological actions of cnidarian venoms and crude extracts is still missing due to the fact that several types of toxins coexist in the same venom. Therefore, it would be important to know not only the effect of a particular toxin but the total effect of the whole venom in vitro and in vivo. Hence, the aim of the present work is to characterize some pharmacological aspects of *Palythoa caribaeorum* venom in terms of hemolytic, antiparasitic and anticancer activities in order to use this organism as a source of new compounds with potential use as candidate drugs.

## Methods

### Laboratory animals

All experiments were performed in accordance with the Official Standard NOM-062-ZOO-1999 for the production, care, and use of laboratory animals. The care and use of the animals was approved by the Bioethics Committee of the School of Medicine, UAQ.

### Venom extraction

*P. caribaeorum* organisms were collected by free diving in La Gallega coral reef in Veracruz, México. The crude extract was obtained according to the method described elsewhere [22]. Briefly, the organisms were carefully separated from the rocks using a chisel and a hammer. In the laboratory, the material was cleaned from remaining rock and soaked in water to eliminate superficial mucus. In order to extract nematocyst venom, the organisms were carefully squeezed in deionized water to expose hidden polyp tentacles and mechanically discharged. The solution was then centrifuged twice at 70,000 g for 15 min at 4 °C, lyophilized, and stored at − 70 °C until use.

### Hemolytic activity assay

The hemolytic assay was performed as described by Rottini et al. [23] with some modifications. Human erythrocyte suspension was prepared from fresh blood from a healthy donor. Blood was collected in a flask with Alsever’s solution buffer (pH 6.4) containing dextrose (0.116 M), NaCl (0.071 M), sodium citrate (0.027 M) and citric acid (0.002 M). The suspension was centrifuged at 2500 rpm for 5 min at 4 °C and the supernatant was decanted. This step was repeated three times and the final pellet was resuspended in Alsever’s buffer. Erythrocytes were incubated at two temperatures 37 °C and 60 °C for 30 min in the presence of different venom concentrations ranging from 1 to 10 mg/mL. Immediately after incubation, the samples were centrifuged at 2500 rpm for 5 min at 4 °C and the optical density of supernatant was measured using a spectrophotometer at 415 nm. The results were normalized to 100% hemolysis by diluting the erythrocytes in deionized water and adjusting the absorbance A_415_ to 0.9 when total lysis occurred.

### Phospholipase A_2_ assay

Phospholipase A_2_ (PLA_2_) activity of the aqueous extract was determined using a secretory PLA_2_ colorimetric assay kit (Cayman Chemical, USA). This assay uses the 1,2-dithio analogue of diheptanoyl phosphatidylcholine as substrate. Free thiols generated upon hydrolysis of the thioester bond at the sn-2 position by PLA_2_ were detected using DTNB [5,5′-dithio-bis-(2-nitrobenzoic acid)]. Color changes were monitored by a Benchmark Plus microplate spectrophotometer at 414 nm, sampling every minute for 10 min. As reference for PLA_2_ activity, 10 μL (10 μg) of bee venom PLA_2_ was used as control. PLA_2_ activity was expressed in μmol of hydrolyzed phosphatidylcholine per minute per mg of protein (*n* = 3).

### Isolated rat aortic ring assay

Male Wistar rats (275–325 g) were anesthetized with chloroform, sacrificed by decapitation and the descending thoracic aorta was removed and placed in ice-cold, oxygenated Krebs-Henseleit solution (126.8 mM NaCl, 5.9 mM KCl, 2.5 mM CaCl2, 1.2 mM MgSO4, 1.2 mM KH2PO4, 30 mM NaHCO3, and 5 mM D-glucose, pH 7.4) and immediately flushed with Krebs-Henseleit solution to prevent intravascular clot formation. The aorta was dissected free of adipose and connective tissue and cut into 4 to 5-mm rings. The aortic rings were mounted between stainless steel hooks and suspended in 7-mL of water-jacketed organ baths containing oxygenated (95% O_2_ and 5% CO_2_) Krebs-Henseleit solution at 37 °C. The tissues were allowed to equilibrate for 60 min under a resting tension of 1.5 g. During this period, the bathing medium was exchanged every 15 min. After final adjustment of the passive resting tension to 1.5 g, aortic segments were contracted with 100 mM KCl.

Once a stable contractile tone was reached, the bathing medium was replaced to restore a resting tension of 1.5 g. After that, the tissues were contracted with 1 μM L-phenylephrine, the force of contraction was recorded, and this contraction was set as 100%. The bathing medium was replaced again to restore a resting tension, and then the extract or the fractions were added to the organ bath. The isometric tension was measured by a Grass FT03 force-displacement transducer attached to a Grass 7D polygraph. The responses were expressed as a percentage of the initial contraction achieved with phenylephrine. The half-maximal effective concentration (EC_50_) and the maximum effect (Emax) values were interpolated by fitting log concentration-response curves (*n* = 3/curve) using non-linear regression analysis.

### Insect toxicity assay

Insect toxicity of the extract was determined by using undetermined sex crickets (*Acheta domestica*) weighing between 200 and 250 mg by a method previously described [24]. Briefly, lyophilized extracts were dissolved in insect saline solution [200 mM NaCl, 3.1 mM KCl, 5.4 mM CaCl_2_, 4 mM MgCl_2_, 2 mM NaHCO_3_, 0.1 mM Na_2_HPO_4_; pH 7.2] and administrated by thoracic injection into crickets (five crickets per dose) at several doses (1, 3.2, 10, 31.6, 100, and 316 μg protein/mL). The injection volume for all crickets, including the controls that received insect saline solution, was 10 μL. Injections were performed using a 0.3-mL gauge insulin syringe (B-D Ultra-Fine, Terumo Medical Corporation, USA). After the injection, crickets were placed in small plastic containers with food and water ad libitum. Mortality was scored at 24 and 48 h post-injection. The lethal dose 50 (LD_50_) values were interpolated by fitting log dose-response curves (*n* = 3/curve) using non-linear regression analysis.

### Cytotoxicity assay

The cytotoxic extract was screened in vitro against human cancer cell lines: HCT-15 (human colorectal adenocarcinoma), MCF-7 (human mammary adenocarcinoma), K562 (human chronic myeloid leukemia), U251 (human glyoblastoma), PC-3 (human prostatic adenocarcinoma), SKLU-1 (human lung adenocarcinoma) and the normal cell lines MT-2 human lymphocytes and J774 rat macrophages. Cell lines were supplied by the National Cancer Institute (NCI, USA). The human tumor cytotoxicity was also determined by using the protein binding dye sulforhodamine B (SRB) in microculture assay to measure cell growth as described in the protocols established by the NCI [25].

The cell lines were cultured in RPMI-1640 medium supplemented with 10% fetal bovine serum, 2 mM L-glutamine, 10,000 units/mL penicillin G, 10,000 μg/mL streptomycin sulfate and 25 μg/mL amphotericin B (Gibco). The cultures were maintained at 37 °C in a 5% CO_2_ humidified atmosphere. With the exception of K-562 and MT-2 cell lines, the rest of the adherent cell lines were removed from the tissue culture flask by adding of 1 mL of 0.05% trypsin-EDTA (GIBCO-laboratories) and diluted with fresh media. The viability of the cells used in the experiments exceeded 95% as determined with trypan blue. For the assay, 100 μL containing 5000–10,000 cells/ well was seeded in 96-well microtiter plates (Costar) and incubated to allow for cell attachment.

After 24 h of incubation, 100 μL of a solution of the test extract obtained by diluting the stocks was added to each well. The cultures were exposed for 48 h to the extract at concentrations of 100 μg/mL. After the incubation period, cells were fixed to the plastic substratum by the addition of 50 μL of cold 50% aqueous trichloroacetic acid. The plates were incubated at 4 °C for 1 h, washed with tap H_2_O and air-dried. The trichloroacetic-acid fixed cells were stained by the addition of 0.4% SRB. Free SRB solution was then removed by washing with 1% aqueous acetic acid. The plates were then air-dried and the bound dye was solubilized by the addition of 10 mM unbuffered Tris base (100 μL). The plates were placed on a shaking platform for 5 min and the absorption was determined at 515 nm using an ELISA plate reader (Bio-Tex Instruments).

### Antiparasitic assay

Antiparasitic activity was performed against *Giardia intestinalis* (WB reference strain, ATCC 30957). Trophozoites were cultured in TYI-S-33 medium in 13 × 100 mm test tubes. When trophozoites were in monolayer (until logarithmic phase of growing), medium was replaced by phosphate buffer (PBS), pH 7.0, cooled in ice for 15 min and centrifuged during 5 min at 3500 rpm. PBS was removed and the trophozoites were counted in a Neubauer chamber. Tests were done in Eppendorf tubes with a final volume of 1.5 mL by using a 50,000 trophozoites/mL of TYI-S-33 medium, and different concentrations (1, 0.5.0, 25, 0.125 and 0.0625 mg/mL) of *P. caribaeorum* extract. Metronidazole (10 μg/mL) was used as positive control. Tubes were incubated at 37 °C for 24 h following by cooling in ice for 15 min and centrifuged. The supernatant was discarded and new medium was added for reculture during for 24 h at 37 °C. Finally, trophozoites were quantified in a Neubauer cell-counter chamber. Percentage of dead trophozoites was plotted against log concentration. IC_50_ and IC_90_ were calculated by graphic extrapolation with JPM 9.0 software.

## Results

### Bioassays

**Fig. 1:**
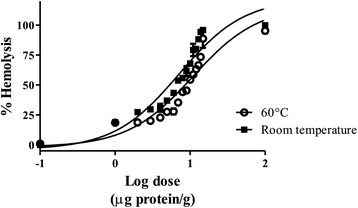
Hemolytic activity of *P. caribaeorum* venom. Human red blood cells were incubated for 30 min at 37° and 60 °C. Values are mean S.E.M. of four independent experiments, with triplicate values

**Fig. 2:**
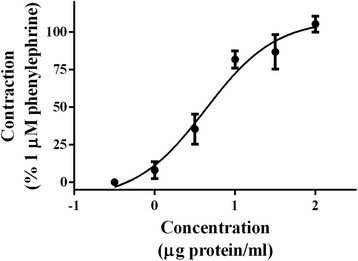
Concentration-response curve showing the vasoconstrictor effect of *P. caribaeorum* venom on rat isolated aorta. Values are expressed as mean ± S.E.M. (*n* = 3). Concentration represents protein content in the extracts

The extract obtained exhibited concentration-dependent hemolytic activity on human erythrocytes. In addition, the activity was reduced, but not abolished, when the extract was incubated in a water bath at 60 °C for 10 min (Fig. 1). It also showed a PLA_2_ activity of 0.155 ± 0.009 μmol/min/mg, while PLA_2_ from bee venom, used as control, displayed an activity of 14.734 ± 0.624 μmol/min/mg. This enzymatic activity was completely lost when the venom was incubated in boiling water bath for 30 min. The induced vasoconstriction on rat aortic rings showed an EC_50_ = 4.287 ± 1.766 with an E_max_ = 108.2 ± 7.167 (Fig. 2).

**Fig. 3:**
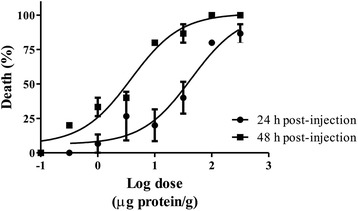
Toxicity of *P. caribaeorum* venom on crickets (*A. domestica*) at 24 and 48 h post-injection

The insecticidal activity results showed that *P. caribaeorum* venom was lethal to crickets, the determined LD_50_ values at 24 h and 48 h for *P. caribaeorum* venom was 50.92 ± 10.85 and 3.78 ± 0.243 μg protein/g respectively (Fig. 3). The venom did not induce immediate paralysis, but at higher concentrations, motility was gradually reduced.

### Cytotoxicity assay

The major inhibitory effect on tumoral cell lines was observed on the glyoblastoma cell line U251 (52.61%), followed by a 41.5% inhibition activity of human lung cancer cells SKLU-1. No significant activity was observed on the rest of the tumoral lines tested. The venom also showed a high inhibition on rat macrophages J774 (53.0%), but slight activity on human T lymphocytes MT-2 (11.01%). No activity was observed against the other cell lines.

### Antiparasitic assay

**Fig. 4:**
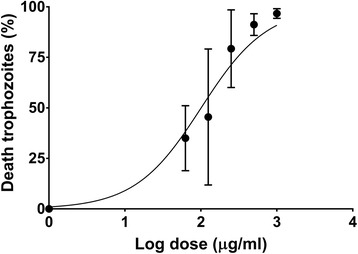
Antigiardial activity of *Palythoa cariboeroum* whole extract

**Fig. 5:**
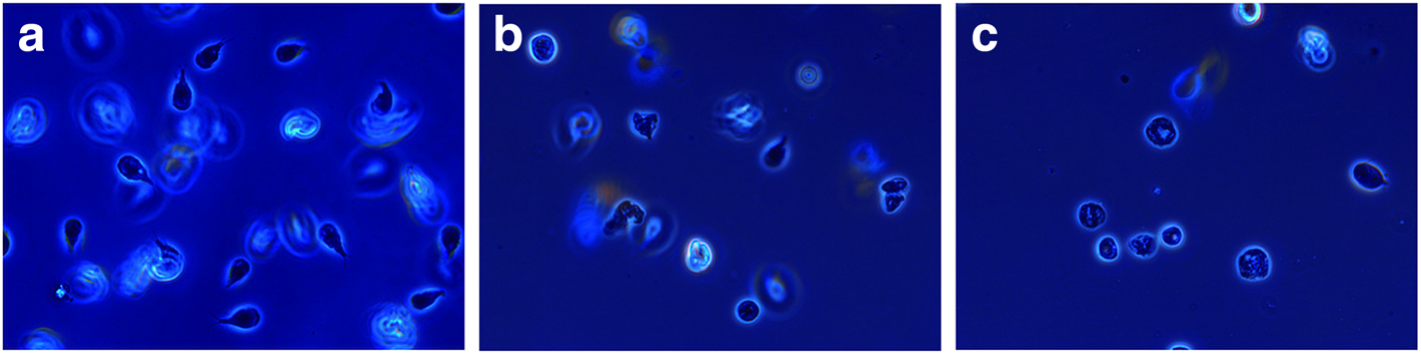
Acivity of *P. caribaeorum* extract on *Giardia intestinalis* trophozoites. **a** Trophozoites without extract, (**b**) trophozoites exposed to 500 μg/mL, (**c**) Trophozoites with 1000 μg/mL of extract

The antiparasitic tests against *G. intestinalis* showed that the extract contains substances capable of killing the parasite in a dose-dependent manner (Fig. 4). The IC_50_ and IC_90_ values were 116 and 603 μg/mL, respectively. These values are high compared to that of metronidazole (IC_50_ = 0.55 μg/mL and IC_90_ = 3.54 μg/mL), however, this is a whole extract. Trophozoites exposed to 500 μg/mL and 1000 μg/mL of the venom showed an atypical morphology: rounded, increased in volume, presence of large vacuoles and even many of them were lysed (Fig. 5). These characteristics suggest that the active substances affect the membrane by a mechanism that affects the osmotic equilibrium and finally lysing the cell.

## Discussion

Animals that produce venom are known for the adverse effects that they may provoke in humans, such as allergic reactions, dermatitis, hemorrhage, intravascular coagulation, necrosis, respiratory failure, etc. For this reason, they have become a source of substances with distinct pharmacological properties, many of them explored in cancer research. In recent years, cnidarian extracts and venoms, especially those from sea anemones and jellyfishes, have been investigated for their pharmacological properties in order to find new molecules with potential therapeutic activity [6].

Cnidarian cytolysins, besides being important factors for envenomation, have been extensively studied in terms of their mechanisms of action and are being recognized as tools for biotechnological and pharmaceutical applications [26]. The hemolytic properties of extracts from many species of cnidarians have been widely reported [6]. It has been reported that cnidarian cytolysins act in two ways: by forming pores in the membrane (known as actinoporins in sea anemones) or hydrolyzing certain membrane phospholipids (phospholipases). These toxins are used by cnidarians for the capture and digestion of prey.

*Palythoa* prey comprise fish larvae and planktonic crustaceans, thus, it is likely that its toxins are active against insects. Some sea anemone toxins have been tested on insect voltage-gated sodium channels and specifically one neurotoxin, CgNa from *Condylactis gigantea*, strongly inhibits the inactivation of the insect voltage-gated sodium channel [27, 28]. In a previous study, we found that the extracts of three scleractinian corals induce toxicity on crickets [29]. In the present study, we found that *P. caribaeorum* extract also showed insecticidal activity with gradual paralysis until death with a major potency than that induced by the scleractinian corals. This activity, as with sea anemones, is consistent with the existence of toxins affecting voltage-gated ion channels. The presence of neurotoxic activity in *P. caribaeorum* venom has already been tested on mammalian neurons, but their specificity on these kind of cells over insect channels remains to be determined [30].

In general, local skin reactions and pain are characteristic in cnidarian envenomation. However, some cases result in systemic symptoms such as increased heart rate and cardiovascular collapse [31]. Up to now, the knowledge on cardiovascular toxicity caused by cnidarian venom is limited. Several studies have reported the presence of vasoconstrictor components in diverse cnidarian extracts [32, 33]. The results of the present study reveal the presence of vasoconstrictor components in the extract of *P. caribaeorum*; however, further studies are necessary to elucidate the chemical characteristics and mechanism of these components.

Since ancient times, animal venoms have been used in traditional medicine to treat several diseases such as cancer. Among these, snake venoms have been the most studied. Several toxins, mainly phospholipases, isolated from snakes have been ascribed as the enzymes responsible for the anticancer effect. In addition, some phospholipases A_2_ are cytotoxic to tumor cells, but devoid of lethality, hemolytic and anticoagulant activities which may be suitable for pharmaceutical purposes [33].

The cytotoxicity of extracts from many species of sea anemones on several cancer cell lines has been reported [34, 35]. Our results showed that the extract specifically inhibits approximately 40% of SKLU-1 human lung adenocarcinoma cells and more than 50% of U251 human glioblastoma. SKLU-1 cell line was reported to be sensitve to the sea anemone *Bunodeopsis globulifera* venom when applied along with cisplatin [36]. According to these results, it may be of great interest to study cnidarian venoms in order to discover molecules that in combination with anticancer drugs may allow the reduction of chemotherapy doses [6].

One of the major causes of human diarrheal diseases, particularly in children, is giardiasis. There are several substances against the parasite *G. intestinalis*, but it is believed that their massive use can result in the development of resistance. Metronidazole is the drug of choice against giardiasis, but is not 100% effective and may produce undesirable side effects such as headaches and metallic taste in the mouth [37]. It has also been shown to be mutagenic and teratogenic in laboratory animals [38, 39]. The search for antiparasitic agents in marine organisms is extensive, however, there are few reports about the effects of venoms from sea anemones and jellyfish against bacteria and parasites [40, 41]. The antigiardial in vitro assays of several cnidarian extracts show good activity of the jellyfish *Linuche unguiculata* (IC_50_ of 63.2 μg/mL) and poor activity of the sea anemone *Stichodactyla helianthus* (IC_50_ of 1388 μg/mL) [16]. Nevertheless, the antigiardial activity was improved when the extract was replaced by a compound obtained from cnidaria [42].

The components responsible for this kind of activity have not been isolated, but according to the morphological changes and final lysis observed in our experiments, we could hypothesize that the molecules involved in this antigiardial effect could be cytolysins and/or phospholipases. The best known cnidarian cytolysins are actinoporins, cytolitic proteins that permeate cell membranes by forming transmembrane pores and causing cell lysis [43]. Although no actinoporin has been isolated from zoanthids, their presence has been well stablished within sea anemones.

*P. caribaeorum* contains phospholipases with potential membrane lysis activity. Actually, a 16 kDa phospholipase A_2_ has been isolated from *P. caribaeorum* but its mechanism of action is still to be elucidated. Finally, another potential mechanism, although not observed within the present study, could be the presence of molecules that elicit morphological changes via the damage of trophozoites cytoskeleton by albendazole or curcumin [44].

Cytotoxins isolated from different venom sources have shown various physiological effects, such as modulation of the activity of membrane enzymes, depolarization of excitable membranes, inhibition of platelet aggregation, cardiac arrest, hemolysis and cytotoxicity [33]. The experiments carried out in this study showed the presence of cytotoxins in *P. caribaeorum* extract. These toxins, although not chemically described here, must be of proteinacious nature. Such hypothesis is based on previously reported mass spectrometry analysis and by the loss of the enzymatic activity after incubation of the extract with boiling water [30]. However, we cannot discard the presence of anticancer terpenoids, since they are abundant and have been isolated in all classes within phylum Cnidaria [6].

## Conclusions

In summary, the present results show that *P. caribaeorum* contains substances with a broad variety of pharmacological activities, which makes the order Zoantharia – including sea anemones and jellyfishes – a viable option in the search for novel molecules. Further research is necessary to identify the molecules that exert these activities and to determine whether the venom contains useful compounds suitable for other pharmaceutical purposes.

## Abbreviations

EC_50_: Half-maximal effective concentration
LD_50_: Lethal dose 50
NCI: National Cancer Institute
PLA_2_: Phospholipase A_2_
